# Thymic Engraftment by *in vitro*-Derived Progenitor T Cells in Young and Aged Mice

**DOI:** 10.3389/fimmu.2020.01850

**Published:** 2020-08-18

**Authors:** Jastaranpreet Singh, Mahmood Mohtashami, Graham Anderson, Juan Carlos Zúñiga-Pflücker

**Affiliations:** ^1^Department of Immunology, University of Toronto, Toronto, ON, Canada; ^2^Sunnybrook Research Institute, Toronto, ON, Canada; ^3^Institute of Immunology and Immunotherapy, University of Birmingham, Birmingham, United Kingdom

**Keywords:** T cell progenitors, T cell development, thymus regeneration, thymic hypoplasia, thymic involution, aged thymus

## Abstract

T cells play a critical role in mediating antigen-specific and long-term immunity against viral and bacterial pathogens, and their development relies on the highly specialized thymic microenvironment. T cell immunodeficiency can be acquired in the form of inborn errors, or can result from perturbations to the thymus due to aging or irradiation/chemotherapy required for cancer treatment. Hematopoietic stem cell transplant (HSCT) from compatible donors is a cornerstone for the treatment of hematological malignancies and immunodeficiency. Although it can restore a functional immune system, profound impairments exist in recovery of the T cell compartment. T cells remain absent or low in number for many months after HSCT, depending on a variety of factors including the age of the recipient. While younger patients have a shorter refractory period, the prolonged T cell recovery observed in older patients can lead to a higher risk of opportunistic infections and increased predisposition to relapse. Thus, strategies for enhancing T cell recovery in aged individuals are needed to counter thymic damage induced by radiation and chemotherapy toxicities, in addition to naturally occurring age-related thymic involution. Preclinical results have shown that robust and rapid long-term thymic reconstitution can be achieved when progenitor T cells, generated *in vitro* from HSCs, are co-administered during HSCT. Progenitor T cells appear to rely on lymphostromal crosstalk via receptor activator of NF-κB (RANK) and RANK-ligand (RANKL) interactions, creating chemokine-rich niches within the cortex and medulla that likely favor the recruitment of bone marrow-derived thymus seeding progenitors. Here, we employed preclinical mouse models to demonstrate that *in vitro*-generated progenitor T cells can effectively engraft involuted aged thymuses, which could potentially improve T cell recovery. The utility of progenitor T cells for aged recipients positions them as a promising cellular therapy for immune recovery and intrathymic repair following irradiation and chemotherapy, even in a post-involution thymus.

## Introduction

T cells are essential mediators of antigen-specific, long-term adaptive immunity. The thymus is responsible for the development of self-tolerant, immunocompetent T cells, but given a lack of self-renewing cells, is continually reliant on replenishment of new T cell progenitors derived from bone marrow (BM) hematopoietic stem cells (HSCs). Subsequent maturation of T cells occurs through a series of tightly regulated and directed differentiation stages that are dependent on signals from the specialized thymic microenvironment. These processes lead to the generation of mature CD4^+^CD8^−^ and CD4^−^CD8^+^ single positive (SP) T cells that exit the thymus and seed the peripheral organs (spleen, lymph nodes), where they can subsequently encounter antigen, undergo expansion, and acquire effector or memory functions (1).

**Figure 1:**
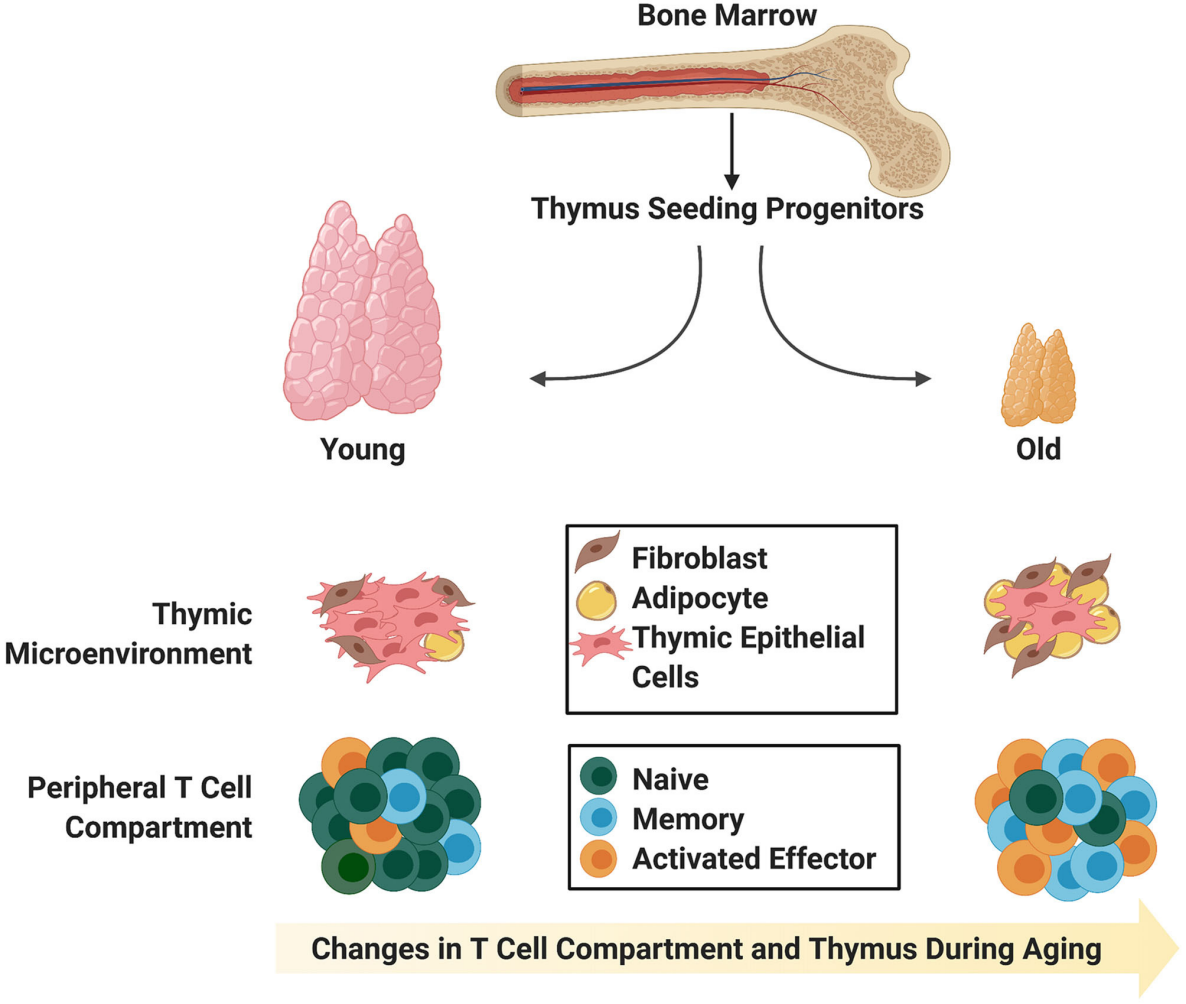
Age-associated changes in the thymic stromal and T cell compartments. The young thymus is mainly comprised of thymic epithelial cells (medullary and cortical; not distinguished) capable of supporting rigorous thymopoiesis of naïve T cells with T cell receptor diversity. Naïve T cells comprise the largest proportion of peripheral T cells in young individuals. In contrast, the involuted aged thymus contains adipocytic and fibrotic cells, and the reduction in thymic epithelial cells and physical changes in thymic morphology do not support robust generation of naïve T cells with T cell receptor diversity. Instead, there is an enlargement in the peripheral memory T cell compartment, which is capable of giving rise to effector T cells upon stimulation.

T cell immunodeficiency, or T lymphopenia, can result from perturbations to the thymic microenvironment due to age-related thymic involution, irradiation required for cancer treatment or from infections that directly target T cells (2). One of the major causes of T cell deficiency is primary immunodeficiency, which is often inherited, leading to an early disease onset. Extrinsic factors can also adversely affect the immune response. In particular, the use of chemotherapeutic agents such as cyclophosphamide prior to hematopoietic stem cell transplantation (HSCT) can cause rapid transient involution of the thymus (3–5). While these drugs are critically required for eradication of the cancer cells, they also harm healthy dividing cells, including cells of the hematopoietic compartment and thymic epithelial cells (TECs) that constitute part of the thymic microenvironment. As a result, there is a delay in T cell recovery following HSCT, and a paucity of *de novo* T cell generation from HSC-derived progenitors (5–9), which can be particularly problematic for aged patients that are concomitantly undergoing age-related thymic involution (Figure 1). The end result is dramatic changes in the T cell compartment of patients including a decline in naïve T cell output, reduced T cell diversity, and increased susceptibility to infection, autoimmune diseases and cancer (10). Therefore, altered thymic architecture is a key trigger for the deterioration of T cell-related immune function in the aged, and insight into strategies that enhance thymic function in adults is of critical importance. Here, we explore the challenges of T cell recovery and thymic regeneration following myeloablative and irradiation treatments, and leading approaches in the field to overcome these issues. We focus on recent advances that take advantage of cell-based treatments, such as progenitor T cell engraftment, for overcoming periods of immunodeficiency following HSCT, particularly in aged individuals.

## T Cell Reconstitution After Myeloablative Treatments

Allogeneic HSCT is a mainstay for the treatment of a large number of diseases of the hematopoietic system. A number of modifications to HSCT procedures, including T cell depletion, CD34^+^ hematopoietic stem/progenitor cell selection, and the use of irradiation and chemotherapeutic drugs, have greatly improved post-transplant clinical outcomes (11, 12). Nevertheless, T cell repopulation post-transplantation remains a major hurdle (5). T cell recovery is often delayed by months and it may take years to fully restore normal numbers of T cells and functionality, if at all (13, 14). Furthermore, there appears to be an inverse correlation between time to post-HSCT T cell recovery and the age of the recipient (15). While it may take up to 6 months to 1 year in the young to recover T cells with a wide T cell receptor (TCR) repertoire, it may take years in adult patients to witness evidence of new T cell generation, if ever (16–18). With an increasingly aging population, there is an imminent need for a dependable way to reconstitute all blood cells, including T cells, in cancer patients that have received chemotherapy and irradiation. Otherwise, patients remain susceptible to a variety of complications that can result in mortality due to general weakened immunity that renders them vulnerable to opportunistic infections and potential cancer relapse (19).

To mitigate cancer relapse, many clinics maintain the standard practice of not depleting donor T cells from HSCT grafts. While donor T cells contained within the graft can mount anti-tumor responses against the relapsed cancer, they can also induce graft vs. host disease (GvHD) against vital organs, even in human leukocyte antigen (HLA)-matched transplants (20). The complications of GvHD are, in turn, managed by defined doses of prescribed immunosuppressants, which aim to balance the anti-tumor effects of the graft with the suppression of GvHD (21). However, this is an unlikely long-term solution for patients, as the vast majority of patients succumb to GvHD or cancer relapse due to immunosuppression within 5 years post-transplant. To this end, an extensive understanding of the phenotypic changes and microenvironmental cues facilitating the transition of HSCs in the BM to T cells in the thymus may be useful for development of strategies to improve T cell outcomes post-HSCT.

### The Intrathymic Intricacies of T Cell Development

T cell precursors arise from HSCs in the adult BM (Figure 1). Thymus seeding progenitors (TSPs) arrive in the thymus in small numbers, where upon interaction with the thymic epithelial cells (TECs), give rise to early thymic progenitors (ETPs). ETPs in both human and mice differentiate through successive CD4^−^CD8^−^ double negative (DN) stages that require Notch receptor and Delta-like-4 (Dll4) ligand interactions. Targeted deletions of either *Notch1* receptor in hematopoietic cells, or *Dll4* in TECs, result in the abrogation of T cell development (22–25). Notch signaling mediates cell fate restriction, which begins within the BM TSP subset, and continues following entry into the thymus (26).

Proper stage-specific maturation of T cell precursors is dependent on thymic structure and the diverse cells present in thymic stroma. The thymus is differentiated into an outer cortical and an inner medullary region, which contain cortical and medullary TECs (cTECs and mTECs), respectively. Their maintenance, in turn, is governed by a complex interplay between lymphoid and stromal compartments within the thymus (termed lymphostromal thymic crosstalk) (27). Studies in the postnatal mouse have demonstrated that TSPs express CCR7 and CCR9, and enter the thymus at the junction between the cortex and medulla (1). Thymic entry is governed by the chemokines CCL21 and CCL25, which are expressed primarily by mTECs and cTECs, respectively. As they differentiate, T cell precursors migrate outwards toward the subcapsular zone of the cortex in response to a CCL25 chemotactic gradient (1, 28, 29). In the subcapsular zone, the CD4^−^CD8^−^ DN thymocytes proliferate and subsequently differentiate into CD4^+^CD8^+^ DP cells that up-regulate CCR7 on their cell surface and migrate toward the medulla (mediated by CCR7 ligands, CCL19 and CCL21) where the final stages of T cell development take place (30). These chemokine-receptor interactions constitute one of the pivotal crosstalk signals dictating strict migration of developing thymocytes through the thymus, and they are conserved in humans (31, 32).

Thymic crosstalk also induces proliferation and ensures selection of a T cell repertoire that is self-tolerant, but reactive to foreign challenges. Relatively little is known of the involvement of crosstalk in the development and function of the thymic cortex, although the notion of crosstalk is well-accepted (27). Mouse models showing a block in early T cell development (CD3ε transgenic mice, CD117^−/−^CD132^−/−^ double-mutant mice) lack demarcation of cortical and medullary areas, and show marked reductions in thymocyte numbers compared to wildtype (WT) mice, implying that the early stages of T cell development are important in TEC maturation (27). Thymic crosstalk during positive and negative selection events have been better defined. Low/intermediate affinity interactions between TCR and major histocompatibility complex (MHC)/peptide complexes on TECs prevent cell death during positive selection. Positively selected thymocytes then localize to the medulla, where high affinity interactions between the TCR and MHC/peptide complexes on thymic dendritic cells result in apoptosis via negative selection of self-reactive cells (1, 28). Self-tolerance by negative selection is established through the expression of the transcription factors autoimmune regulator (*Aire*) and *Fezf2* by mTECs (33). These two independent factors are regulated by distinct signaling pathways, the RANK/CD40 pathway and the LTβR pathways, respectively, are non-redundant and promote the expression of peripheral tissue-restricted antigens (TRA) on mTECs (33, 34). T cells displaying a strong self-reactivity toward TRA are deleted.

The non-canonical nuclear factor-κB (NF-κB) pathway has a well-established role in thymic crosstalk during selection. Lymphotoxin-β receptor (LTβR), RANK, and CD40 are the key tumor necrosis factor (TNF) superfamily members that signal through the non-canonical NF-κB pathway in the context of thymic crosstalk and self-tolerance (35). In mice, LTβR deletions led to defective medullary development, and a reduced or absent medulla. The reduction of Aire^+^ mTECs was relatively mild (35, 36), although Fezf2-dependent TRA expression on mTECs was drastically decreased (33). The involvement of CD40 ligand (CD40L)-CD40 interactions has also been described (35). Overexpression of CD40L in the thymus resulted in abrogated thymus cortex formation and medullary expansion, whereas analysis of CD40L-deficient mice revealed no obvious defect in medullary organization by immunohistochemistry. RANK- and RANKL-deficient mice exhibit a severe reduction in Aire^+^ mTECs and overall mTEC numbers, although the formation of a thymic medulla is still detectable. Furthermore, RANKL-RANK and CD40L-CD40 signaling exhibit a cooperative role in mTEC development and self-tolerance (35). Notably, the defects seen in these deficient mice do not affect Fezf2-restricted TRA expression and are milder compared to mutant mice deficient in the NF-κB activation pathway such as *aly/aly* (NF-κB-inducing kinase mutation), and RelB-deficient mice (35–37). Although the expression and function of these molecules on thymocyte and TEC subsets is well-understood in mice, this has yet to be elucidated in the human context. Furthermore, harnessing insights from these developmental processes could be important for restoring the thymus and T cell-mediated immunity in the aged following HSCT.

## Age-Related Thymic Involution

Defects in both the hematopoietic and thymic stromal components contribute to decreased thymic output in aged individuals. The fundamental reason for the late or absent T cell recovery in aged patients is the natural and progressive decline of the immune system, especially of the thymus, in a process called thymic involution. Thymic involution starts to occur before puberty, but is at its peak during puberty and the decline continues steadily with age (38). Although thymus involution in the aged was noted many years before the function of thymus was discovered (39), the evolutionary advantage of this physiological process remains a mystery. Thymic involution, which results in its several fold size reduction, is not perceptible with physical examination, but it is concomitant with physiological changes that can be measured. Multidetector computed tomography (CT) scans can detect increasing replacement of soft tissue that is mainly occupied by thymocytes and TECs by a less dense, X-ray permissive fatty tissue (40, 41). These changes impact both the thymocyte and TEC compartments of the thymus, having detrimental impacts on the establishment of T cell-based immunity in the adult.

### Impairments in T Cell Development

Since the thymus requires continuous seeding by BM HSC-derived progenitors, the chance of T cell generation is further diminished since aged HSCs have a propensity to differentiate toward the myeloid fate (42) in both mice (43–47) and humans (48, 49). These alterations in HSC function inevitably affect the lymphoid compartment, and different groups have shown that aged mice not only have less ETPs, but that the ETPs also have reduced expansion and differentiation potential (50, 51). Furthermore, HSCs from old mice do not efficiently repopulate lymphoid cells in young recipients (46).

The later stages of T cell development are also affected by aging. DP and SP thymocytes in mice show reduced CD3 expression, suggesting that they may have potential defects in TCR-based stimulation (52–54). These results are supported by the observation that cells from aged mice have cell cycle defects (53). It follows that defects during development could impact the kinetics, proliferation, signaling capacity, and therefore, immunocompetence, of recent thymic emigrants (RTEs) from the aged thymus (55, 56). It has been demonstrated that the size of the peripheral T cell pool remains relatively consistent throughout life, even after thymic involution (57), so it is likely that these defects are etched into RTEs during their development within the thymus. RTEs can be detected through assessment of T cell receptor excision circles (TRECs) (58) or by cell surface markers in T cells circulating in the blood (59, 60). Studies have shown that there are lower TREC levels in elderly individuals, and these are associated with an ~80% reduction in naïve T cells (61–64) and an increase in the memory T cell compartment (Figure 1). The memory T cells in adults, while able to undergo proliferative responses, possess defects including skewed cytokine production (65, 66).

Taken together, there are several immunological consequences of aging including the continual decrease in naive T cell output, a limited TCR repertoire (67) and hence, the narrowing of the diversity of foreign antigens potentially recognized by aging individuals, leading to a higher susceptibility to pathogens.

### Changes in the Thymic Stromal Microenvironment

Age-induced changes within the thymic niche could likely account for many of the T cell developmental defects seen within the aged thymus. During thymic involution, there are alterations in the architecture starting at around the time of puberty when the rate of atrophy is greatest (38, 68, 69). This atrophy is concomitant with a blurred demarcation between cortical and medullary regions, down regulation of various TEC markers, and increased adiposity (70, 71) and fibrosis (72, 73) of the aged thymus (Figure 1). Increased adiposity, particularly in the human thymus, has been linked to an inhibition of thymic function (71). In support of this notion, one group demonstrated that induction of obesity in a mouse model accelerated thymic involution (74), and further, inhibition of adipogenesis within the thymus could ameliorate age-related thymic involution (70). The mechanism behind how adipocytes within the thymus alter T lymphopoiesis and thymic function are unclear, but likely involve the action of cytokines secreted by adipocytic cells, which aggravate, but do not cause, thymic involution (10, 71). Given the impairments in both the thymocyte and TEC compartments during age-related thymic involution, it could be expected that lymphostromal crosstalk between these two compartments is also impaired and is a likely contributing factor toward thymic involution.

### HSCT in the Aging Population

Considering the typical disadvantages presented by the aged thymic microenvironment, further injury by conditioning regimens to susceptible TECs could be critically damaging to thymic engraftment following HSCT (75, 76). There is a substantial lag between the time of bone marrow engraftment following HSCT and migration of thymic seeding progenitors to the thymus even in younger patients. Preclinical studies confirmed that total body irradiation followed by HSCT results in poor engraftment in the aged thymus when compared to young (73). During this refractory period, there is an absence of crosstalk between thymic stromal and hematopoietic cells, which prolongs thymic regression. The resulting inferior stromal environment is inefficient for T cell proliferation of even young thymocytes (77). It also leads to blurred definition of boundaries between the medullary and cortical regions, suggesting inadequacies in TEC function (72). In support of this notion, the age-related decrease in Aire expression in the medulla and abnormally low negative selection of emerging thymocytes, led to increased autoreactivity and inflammation (78). Taken together, there appears to be a confluence of events that undermine the restoration of conditions for *de novo* generation of T cells within the aged thymus following HSCT. Employing strategies that aid in thymic regeneration and T cell reconstitution remains an important task.

## Strategies for Enhancing Thymic Regeneration

To reverse the disadvantages posed by age-related changes, there have been wide-ranging attempts to protect the thymus from the deleterious effects of myeloablative regimens, and preserve and strengthen thymic properties that enable T cell reconstitution. While there are many lines of evidence for the demise of the thymus with age, there is confirmation of its continued function as an essential component of immunity. Detection of TRECs has confirmed that there is a constant output of naïve T cells or RTEs that continue to migrate to the periphery (8). There is also strong evidence for recruitment of TSPs into the thymus after HSCT (73). Comparison of the young and the aging thymus has led to the discovery of a range of possible interventions that aim to improve the thymic milieu to rejuvenate the stromal cells. These include keratinocyte growth factor (KGF) administration for protecting TECs during irradiation-induced injury (79–82), and IL-22 administration for stimulation of TEC proliferation and repair (83). However, these approaches have not led to rapid, early or complete restoration of the peripheral T cell compartment after transplant, and it is difficult to delineate whether these strategies directly enhance thymic activity in the aged, or, in part, due to the promotion of hematopoiesis in the BM. Furthermore, these approaches focus solely on restoration of the thymic microenvironment, but do not address the effects on progenitor cells incoming to the thymus.

Accordingly, the most remarkable effect on thymus size and output was uncovered as a consequence of physical (84) or chemical castration in both sexes (51, 85), revealing the potential for reversal of age-related thymic atrophy. These effects may be blocked by administration of sex hormones, demonstrating the direct effect of androgens on immunity, and specifically, on thymus involution. Chemical castration involves the administration of agonists of gonadotropin-releasing hormone receptor, which eventually leads to *hypo*gonadism and hence, a reduction of sex hormones, testosterone and estradiol, a treatment also known as sex steroid inhibition (SSI). SSI results in physiological changes in the thymus at the molecular level, including an increase in the chemokine CCL25 for the recruitment of CCR9-bearing progenitor cells (86, 87). Importantly, SSI also appears to directly increase DLL4 expression in TECs (88), which is typically downregulated in the aging thymus. Age-related perturbation of DLL4 expression levels appear to be restored by SSI (88), enabling effective inhibition of B cell development and promotion of mainly T cell development, but also generation of alternative lineages such as dendritic cells (89).

The effect of thymic restoration by SSI, while striking, is transient, with the mouse thymus reaching its peak within 2 weeks of treatment and returning to its involuted size after 2 weeks (68, 90). In addition, although cellularity within the thymus is temporarily restored, aged TECs, especially in the medulla, remain qualitatively different from those from a young thymus according to one study (68). Gene expression profiles of aged TECs restored by SSI largely aligned with the TECs from non-castrated control aged mice and not the young mice. Furthermore, there was limited TRA presentation by regenerated TECs, which created an imbalance in central tolerance. Despite this shortcoming, no adverse effects were observed in SSI-treated preclinical models, although there is evidence or speculation for regulatory T cells mitigating autoreactivity by cells that may have escaped central tolerance. At this point, it appears that the protective benefits of creating more RTEs that mature in the periphery may outweigh the adverse effects of potential autoreactive T cells that escape negative selection processes (68, 91). Hence, SSI remains a viable option for treatment of the aged to generate *de novo* peripheral T cells. In this regard, when SSI treatment was given following the combination of chemical therapy and HSCT in preclinical models, thymocyte recovery was twice as efficient with SSI treatment (85).

In contrast to the clear increase in the number of thymocytes after cytoablative treatment followed by SSI, the TEC compartment of the thymus appears to increase in cell size, but not necessarily due to TEC proliferation (85, 92). Elegant immunofluorescence analyses of TECs demonstrated that morphological changes in TECs, and not *de novo* regeneration, were largely responsible for the expansion of the cortical region of the aged thymus after SSI treatment (92). Concurrently, genetic pathways associated with cell morphology exhibited the most dynamic changes in TECs during thymic regeneration.

Interestingly, the plasticity observed in the thymus is not unique to SSI treatment. In many diseased states, including lymphocytic choriomeningitis virus (LCMV) infection as an example, the thymus is diminished in size. Consistently, a dramatic reduction in thymocyte number (especially the CD4^+^CD8^+^ DP population) was observed (93). This is likely a protective measure against potential tolerization to viral antigens. The observation that the thymus is reduced to a fraction of its normal size within 9 days after LCMV infection, and the restoration to its former size 15 days after the virus is cleared, is reminiscent of the thymus' response to SSI treatment. It also suggests that the regenerative ability of the thymus is intrinsic to the thymic stroma.

While the approaches discussed so far have inherent benefits, they do not successfully address the two-pronged problem with T cell recovery after HSCT in aged populations – that is, the need for increased thymus-seeding progenitors in the host, and an intact thymic environment for provision of appropriate developmental cues for T cell development.

## Progenitor T Cells and Thymic Reconstitution

We and others have shown that another promising strategy to enhance T cell reconstitution post-HSCT is the adoptive transfer of *in vitro*-derived proT cells to rapidly restore the T cell compartment and T cell mediated immunity (94–97). Zakrzewski et al. initially demonstrated that adoptive transfer of *in vitro*-derived mouse CD4^−^CD8^−^ DN cells together with HSCs led to increased T cell reconstitution in both the thymus and periphery of mice that had undergone allogeneic HSCT. More importantly, *in vitro*-derived T cells exhibited early graft vs. tumor activity in HSCT recipients (95). We extended this to humans and characterized a CD34^+^CD7^++^ proT cell subset generated from umbilical cord blood (UCB)-HSC co-cultures with OP9-DL1 cells (96). This population was capable of homing to and engrafting the thymus of NOD-*scid* IL2Rγ^null^ (NSG) immunodeficient mice, demonstrating their ability to “kickstart” the process of T cell recovery. The human proT cell population could be further fractionated based on CD5 expression, with CD5^+^ proT2-cells having enhanced thymic reconstitution capacity when placed in competition with CD5^−^ proT1-cells. Of note, human proT2-cells, when transferred together with HSCs, had the ability to facilitate long-term HSC-derived T-lymphopoiesis in a preclinical mouse model (97). Thus, the thymus-bound potential of proT cells offers several advantages for their implementation during HSCT by shortening the time a patient is left immunocompromised.

Importantly, proT cells lack a cell-surface TCR and therefore are unable to induce GvHD. Strict histocompatibility between proT cells and the host is not required (2, 98), as developing T cells within the thymus undergo selection and tolerization by the host thymus. After proT cell adoptive transfer in pre-clinical mouse models, the mature functional T cells that emerge have a broad repertoire capable of combatting both cancer and infections (95, 99), and can develop into various functional subsets (effector, helper, memory) (100), while demonstrating no autoreactivity.

### A Role for ProT Cells in Lymphostromal Crosstalk

ProT cell-engrafted NSG mice also display thymic plasticity and restoration of the thymic microenvironment (97). We previously demonstrated that the highly disorganized thymic structure of NSG mice was alleviated after adoptive transfer of *in vitro*-derived human proT cells, which induced the organization of cortical and medullary environments (97). These findings were strengthened by the observed increase in transcripts for *Ccl25* and *Ccl19* within the thymuses of proT-engrafted mice. Together, these findings suggested that human proT cells could directly act on stromal elements in the NSG thymus, engage in lymphostromal crosstalk, and thus, promote the formation of chemokine-rich niches for the recruitment of CCR7- and CCR9-expressing BM-derived TSPs.

**Figure 2:**
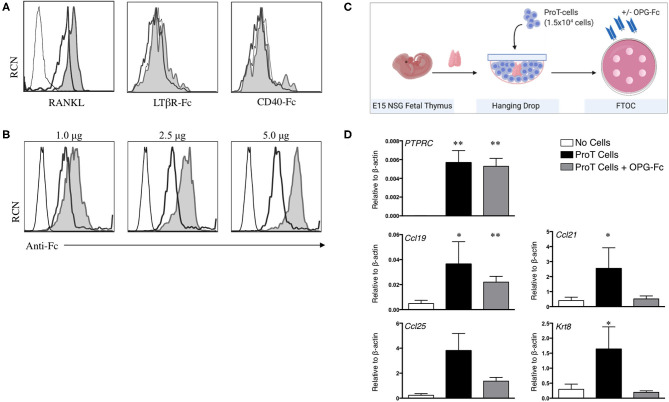
RANKL expression on *in vitro*-derived human proT cells is potentially involved in lymphostromal crosstalk with the thymic stroma. **(A)** Flow cytometric analysis for RANKL, LTαβ and CD40L expression on day 11 *in vitro*-derived CD34^+^CD7^+^-gated *in vitro*-derived proT1 (open, thick line) and proT2 cells (shaded). IgG controls are included as a control (open, thin line). These results are representative of >3 independent experiments for RANKL and LTαβ and 2 independent experiments for CD40L. **(B)** Flow cytometric analysis of 2 × 10^5^ day 10 *in vitro*-derived CD34^+^CD7^+^-gated proT cells showing OPG-Fc staining (shaded), IgG control (open, thick line) and unstained cells (open, thin line). OPG-Fc was used at the doses indicated. **(C)** OPG-Fc was used to block RANK-RANKL interactions in fetal thymic organ culture (FTOC). A schematic of the experimental set-up is shown. **(D)** Gene expression analyses from day 5 NOD/SCID/γc^null^ FTOCs. QPCR analysis for the expression of human *PTPRC* (CD45), and mouse *Ccl25, Ccl19, Ccl21*, and *Krt8* (Cytokeratin-8) for lobes containing no cells (*n* = 8), proT cells (*n* = 7) or proT cells with OPG-Fc (*n* = 7). Transcript levels for all genes were normalized to mouse β-actin. The results shown are representative of at least 3 independent experiments. Statistical analysis was performed using the Kruskal-Wallis one-way analysis of variance with a calculated significance level of *p* > 0.05, followed by *post-hoc* Dunn's test. All data are represented as mean ± SEM, with asterisks representing statistical significance as compared to the control (no cells) group (*p < 0.05, **p < 0.01).

As described above, signaling through the LTβR, CD40 and RANK receptors on TECs are among the key crosstalk signals required for the maturation of the TEC compartment (35, 101). Thus, we assessed cell surface expression of ligands for these receptors (RANKL, LTαβ, and CD40L) on human *in vitro*-derived proT cells (94). We previously reported the expression of RANKL on proT cells, with the proT2 subset expressing 2.5x higher levels than proT1 cells (97), and as shown in Figure 2A. However, we observed little to no expression of LTαβ and CD40L on proT cells. These findings suggest a potential specific role for RANKL in proT cell-mediated effects in the thymus.

As our findings described so far were consistent with the known critical role for RANKL in thymus organogenesis, organization and repair (27), our next goal was to demonstrate a role for RANKL in proT cell-induced effects within the thymus. The naturally-occurring decoy receptor for RANKL, osteoprotegerin (OPG) has a high affinity for RANKL, and is produced physiologically to prevent RANK/RANKL interactions (102). We therefore utilized an OPG-Fc chimeric fusion to block RANKL on proT cells from human HSC/OP9-DL cell co-cultures. OPG-Fc binding of proT cells was dose-dependent, with 5.0 μg of OPG-Fc being the saturating dose for 2 × 10^5^ cells (Figure 2B). Next, we investigated the effects of OPG-Fc-mediated blocking on thymic stroma using fetal thymic organ cultures (FTOC). ProT cells were incubated with saturating doses of OPG-Fc and then used to reconstitute E15 NSG fetal thymus lobes in hanging drop (Figure 2C). ProT-only and non-reconstituted lobes were used as controls, and all lobes were analyzed by QPCR. After 5 days in FTOC, proT-only FTOC cultures revealed an increase in transcript levels for TEC-derived chemokines including *Ccl19* and *Ccl21* compared to non-reconstituted lobes, as expected from our previous *in vivo* observations (97) (Figure 2D) *Ccl25* transcripts were also increased, although the results did not formally reach statistical significance. In contrast, transcript levels for these chemokines, with the exception of *Ccl19*, were not elevated in FTOC cultures containing OPG-Fc. Notably, the presence of OPG-Fc did not affect the ability of proT cells to reconstitute fetal thymic lobes as compared to proT-only FTOC, as the expression levels for the human CD45 transcript (*PTPRC*) were comparable between the two conditions.

While DN thymocytes in mice are known to influence cortex formation, studies have demonstrated that mouse DP and SP thymocytes induce formation of the medulla and maturation of mTECs (103, 104). Specifically, positively selected thymocytes have been shown to regulate the development of the medulla through RANK/RANKL interactions (104). However, it is important to note that our studies reveal a potential role for RANKL on T cell subsets prior to positive selection.

**Figure 3:**
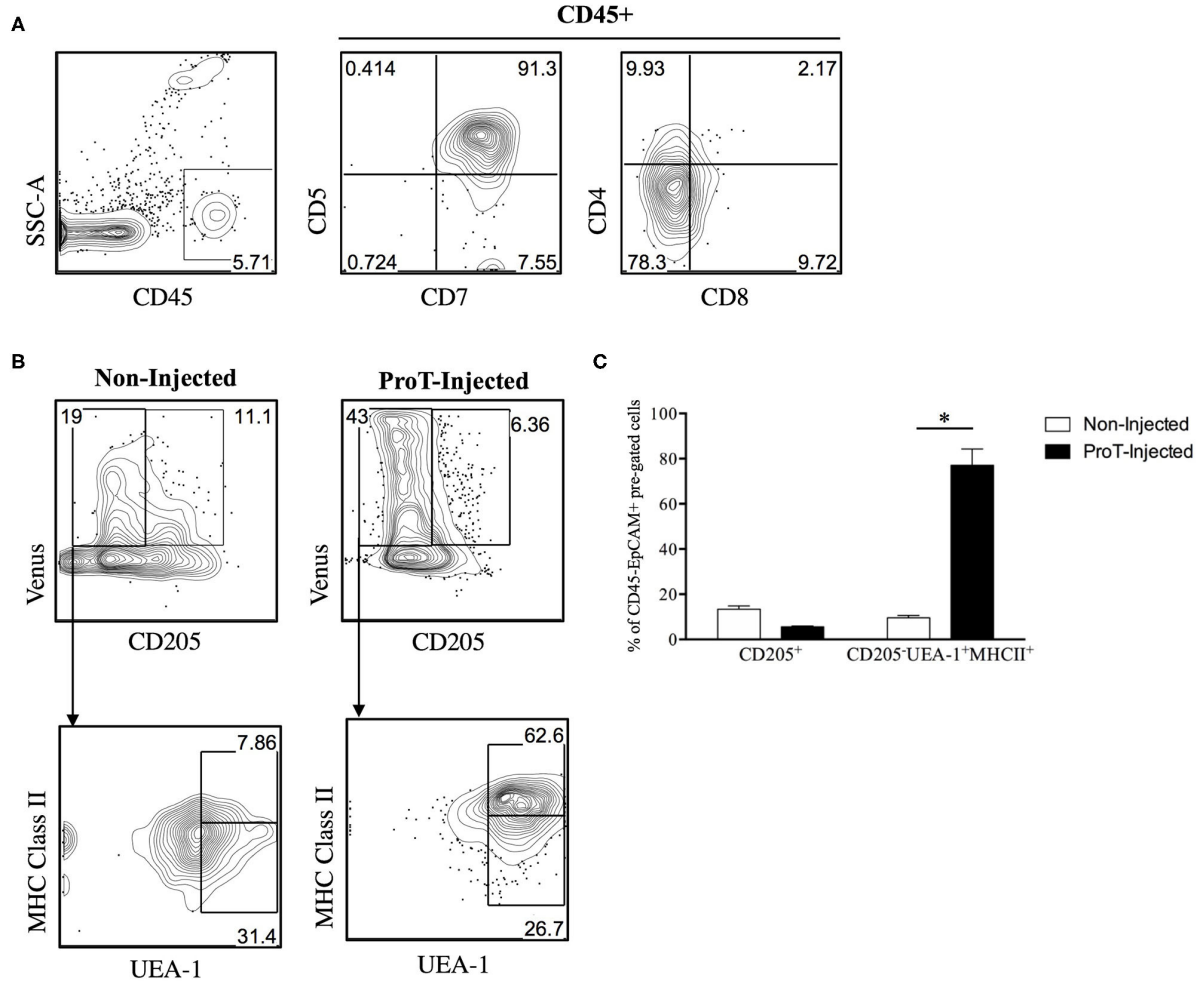
Venus reporter expression in the TEC compartment of immunodeficient RV-SRG mice. Thymic epithelial cells were isolated (108) and analyzed from 2-week old, non-injected RV-SRG mice, or RV-SRG mice that were neonatally injected with proT cells (*n* = 3–4 mice per group). RANK Venus^−^ mice that were non-injected or proT cell-injected were used for gating controls. **(A)** Flow cytometric analysis of CD45, CD7, CD5, CD4, and CD8 for detection of thymic engraftment 2-weeks after proT cell adoptive transfer. **(B)** Flow cytometric analysis of CD45^−^EPCAM^+^ pre-gated TECs. CD205 was used to define cTECs or bipotent TEC progenitors, while mTECs were broadly defined as MHC Class II^+^ UEA-1^+^ (107). **(C)** Percentage of CD45^−^EPCAM^+^RANK Venus^+^-gated CD205^+^ cells or CD205^−^UEA-1^+^MHCII^+^ cells in non-injected or proT-injected mice. Asterisks indicate statistical significance between groups and data are plotted as mean ± standard error of the mean (SEM) (*p < 0.05, two-tailed unpaired student's *t*-test).

Little is known about the role of the RANK/RANKL axis in facilitating lymphostromal crosstalk by DN thymocytes, including proT cells. In a study of mouse thymocyte subsets, Hikosaka et al. noted that DN cells express high levels of RANKL transcripts, while they fail to express LTα, LTβ or CD40L (35). Developmentally, this subset is comparable to the human proT cell population. Consistently, it was also reported that a small subset of cells within the mouse cortex, a proportion of CD205^+^CD40^−^ cTEC-restricted progenitors (105) express RANK (106). To further elaborate on the potential role of RANKL within our proT cell transfer model, we generated an immunodeficient version of the RANK Venus reporter mouse (107), referred to as RANK-Venus hSirpα^Tg^Rag^−/−^γc^null^ (RV-SRG) mice. We showed that proT cells were capable of thymic engraftment in RV-SRG mice, with the emergence of CD45^+^CD7^+^CD5^+^ cells 2-weeks post-injection (Figure 3A). Importantly, these donor-derived cells were not yet at the DP stage, allowing us to focus on events mediated by DN cells. Analysis of the TEC compartment in control (non-injected) RV-SRG mice demonstrated a sizeable proportion of cells (11.1%) that were CD205^+^Venus^+^, which is a subset that likely contains immature, bipotent TEC progenitors (106) (Figure 3B). In contrast, proT cell-injected RV-SRG mice displayed less Venus^+^CD205^+^ bipotent TEC progenitors, but instead showed the appearance of more differentiated MHCII^+^ UEA-1^+^ mTECs in the Venus^+^CD205^−^ compartment (Figure 3C). This finding is consistent with the ability of proT cells to induce changes in the thymic microenvironment, including the formation of the medullary compartment, in immunodeficient mice (97).

The generation of the RV-SRG mouse model will enable further characterization of RANKL interactions with RANK-expressing TECs. Importantly, there is known cross-reactivity of human RANKL with mouse RANK (109), providing further support for our observations. Venus^+^ cells could be sorted to assess signaling events, such as the initiation of the downstream components in the non-canonical NFκB pathway (36). Furthermore, *in vivo* analyses, including those with OPG-Fc would be necessary to further evaluate whether RANKL-based signaling is the sole mechanism underlying thymic changes. Later time points can also be assessed to establish whether proT cells and their downstream progeny can drive a fully functional thymic microenvironment including AIRE expression on mTECs. Nevertheless, these findings are consistent with a critical role for proT cell-mediated enhancement of thymic reconstitution, and this is likely due to, in part, a RANKL-induced differentiation and reorganization of the thymic architecture, which leads to more effective recruitment of BM-derived T lymphocyte progenitors after HSCT.

### ProT Cells for an Aged Thymus?

**Figure 4:**
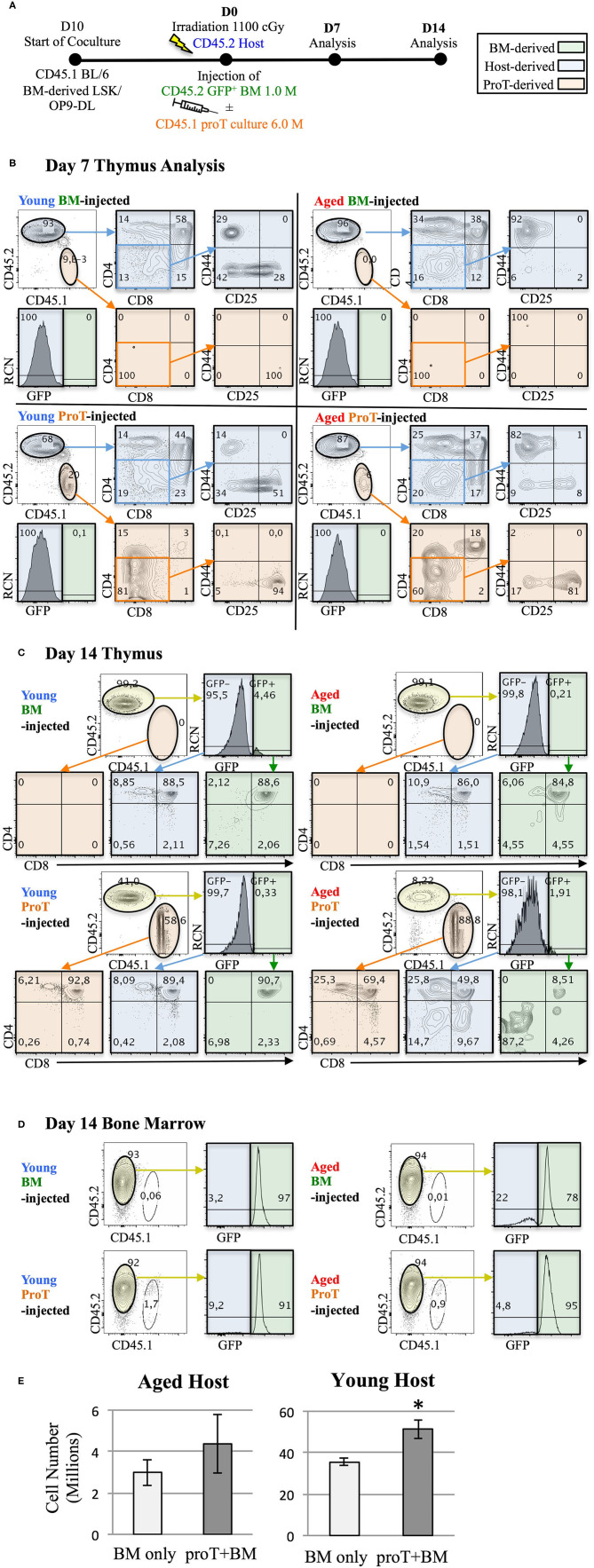
Adoptive transfer of mouse proT cells into lethally-irradiated aged mice. A schematic for experiments is shown **(A)**. LSK/OP9-DL co-cultures were initiated 10 days prior to injection. 18-month old (Aged) and 3-month old (Young) wild-type C57BL/6 (CD45.2) mice were irradiated at 1.025 Gy on the day of injection. All irradiated mice were intravenously injected with 1 × 10^6^ cells extracted from GFP^+^ BM. In addition to the bone marrow cells, some mice also received 6 × 10^6^ proT cells harvested from 10 day co-culture of OP9-DL/BM LSK cells from congenic mice (CD45.1^+^) (*n* = 3 or 4 per group). Plots show the analysis of thymuses that were dissected on 7 **(B)** and 14 days **(C)** after injection. Thymocytes were stained with the appropriate cell lineage markers and analyzed by flow cytometry. Plots showing host-derived cells (CD45.2) are shaded in blue, proT cell-derived cells (CD45.1) are shaded in orange and BM graft-derived GFP^+^ cells are shaded in green. Bone marrow analysis of the corresponding mice in C show contribution of GFP^+^ BM grafts to the BM of the irradiated host on D14 **(D)**. Total thymocyte number was analyzed on D14 **(E)**. The error bars correspond to SEM (*p < 0.05, two-tailed unpaired student's *t*-test).

The notion that proT cells dually serve as TSPs and thymus reorganizers raises the exciting possibility that proT cells could be used as a cell-based strategy to reverse thymic involution. In Figure 4, we show that despite age-related thymic atrophy, mouse proT cells readily engraft the thymus of young and aged mice. ProT cells in mice are contained within the CD25-expressing DN2 and DN3 subsets, both of which have thymic engraftment capacity (110). We co-cultured CD45.1 BM-derived Lineage^−^ Sca1^hi^ cKit^hi^ (LSK) cells with OP9-DL cells for 10 days (Figure 4A). Hematopoietic cells from co-cultures were harvested and enriched for CD25^+^ proT cells. ProT cells were intravenously injected along with BM cells (GFP^+^ to distinguish from host) into lethally irradiated, congenic CD45.2 wild-type (WT) C57BL/6 young (3 months) and aged (18–20 months) hosts. Additionally, young and aged recipients were injected with BM cells only, without proT cells as controls. On D7 and D14, thymuses were harvested and analyzed by flow cytometry.

Irrespective of the age of the host or the cell type injected, a significant portion of differentiated thymocytes was CD45.2^+^ and GFP^−^, signifying that these thymocytes were of host origin. This was consistent with previous observations, which identified radio-resistant host DN cells capable of repopulating the post-irradiation thymus due to niche availability (111, 112). On D7, we observed successful proT cell engraftment in the thymuses of both young and aged mice. However, the vast majority of thymocytes were of host origin (Figure 4B). Of note, the host thymocytes were at the DP, or CD4 and CD8 SP developmental stages compared to the engrafted proT cell-derived thymocytes, which were predominantly at the DN stages, suggesting that DN cells derived from engrafted proT cells were developmentally poised to mature into DP cells. Indeed, by D14, proT cell-derived thymocytes comprised the majority of cells in both aged and young mice (Figure 4C). ProT cells developed into mature T cells and appeared to only slightly lag developmentally compared to the host cells. Therefore, our findings revealed that under these conditions, proT cells contributed to the thymic make-up of both young and aged mice, but did not lead to accelerated thymic reconstitution.

Proportionally, small numbers of GFP^+^ cells derived from the BM graft appeared in the thymus and only by D14 (Figure 4C). This was despite the fact that donor GFP^+^ cells consistently made up over 70% of the cells in the BM (Figure 4D). Notably, while 6 × 10^6^ proT cells were injected compared to 1 × 10^6^ of BM cells, only up to 2% of cells in the BM were proT cell-derived. This was likely a result of the impure CD25^+^-enriched fraction, which contained traces of CD11b^+^ myeloid cells, that were injected into mice (not shown). As such, the CD45.1^+^ cells present in BM were mostly CD11b^+^. We are conducting further experiments to determine whether the contribution of GFP^+^ donor BM-derived cells to the thymus could increase with time. Since both the engrafted proT cells and the host DN cells are not self-renewing, thymus seeding progenitors from the BM would be recruited to fill the niches emptied after maturation of the proT cell graft and the host DN cells.

ProT cell engraftment was successful in both young and aged mice, although we observed a 10-fold difference in the number of thymocytes between young and aged hosts (Figure 4E). Considering that donor BM GFP^+^ cells contribute only up to 5% of the thymocytes, the comparison essentially measured the contribution of proT cells to thymic reconstitution. Importantly, there was an increase in the number of thymocytes with the administration of proT cells in young mice at D14, while no difference was observed in aged mice, despite favourable engraftment by proT cells. Therefore, while the capacity for engraftment of progenitors was high in both the young and the aged hosts, it is likely that the capacity of progenitors to proliferate was better accommodated in young hosts.

Our results show that the injected congenic proT cells were able to compete for the available niches to engraft the thymus of young and aged mice. The kinetics of proT cell-derived thymocytes suggests that proT cells are able to occupy these niches as early as D7, albeit at relatively small proportions. The percentage increase by D14 and increase in cell number suggests that upon reconstituting the thymus, proT cells help to restore thymic activity. It is important to note that the conditions used did not fully mimic cytoablative regimens used in the clinic, which includes successive rounds of chemotherapy (113). Successive rounds of chemotherapy could potentially clear the thymus of the radio-resistant DNs that persist after irradiation. This would render the thymus more similar to the NSG thymus, where proT cells were able to accelerate thymic reconstitution when compared to injection of HSCs alone (97). Further investigation on the addition of chemical therapy in our protocol is underway to closely follow clinical practices and determine whether proT cells “kickstart” the process of thymic reconstitution, particularly now that we have observed that proT cells can engraft involuted irradiated thymuses.

While we show success in engraftment of proT cells in aged mice, further exploration is required for adopting proT cell therapy as part of future clinical trials. Despite the increase in the number of T cells in aged mice using SSI, there is evidence that RTEs are blocked from entering the lymph nodes and functioning effectively (114). Age-related changes in the physiology of lymph nodes may render them incapable of recruiting T cells. Therefore, a disease model showing the effectiveness of the newly generated T cells will be important to demonstrate improvements in functional immunity. In addition, mouse-derived OP9 cells are not appropriate for human or clinical use and alternative systems that are amenable to Good Manufacturing Practices (GMP) would be required. Recent progress and breakthroughs have led us from OP9-DL cells to a two-dimensional (2-D) plate-bound matrix with DLL4 to readily generate human (CD34^+^CD7^+^) and mouse (DN CD25^+^) proT cells that are able to engraft mouse models (115, 116). The next step would be to expand the human culture system to large scale in order to generate sufficient number of proT cells to successfully engraft the thymus of patients (98).

## Conclusion

The targeted approaches with proT cells, cytokine and SSI treatments, all exploit different non-overlapping pathways to improve thymic engraftment and function. ProT cells appear to engage in crosstalk with TECs through RANK/RANKL interactions. Cytokine-specific interventions may activate various signaling pathways, and SSI lifts the suppression imposed by sex hormones on the thymic environment. Ideally, a combination approach where cytokines, SSI and proT cells are cooperatively administered could potentially have additive effects on reversing the damaging effects of cytoablative treatments on thymic function. In this scenario, cytokines would potentially protect TECs from chemical and radiation-induced damage, while injected proT cells could fill receptive niches made available through the morphological thymic changes induced by SSI. This combination strategy has the potential to be adapted to improve T cell-dependent immunity in aged populations.

## Data Availability Statement

The raw data supporting the conclusions of this article will be made available by the authors, without undue reservation.

## Ethics Statement

All animal studies were reviewed and approved by Sunnybrook Research Institute Animal Care Committee. Human UCB samples were obtained in accordance with approved guidelines established by the Research Ethics Board of Sunnybrook Health Sciences Centre.

## Author Contributions

JS designed and performed RANK/RANKL experiments and *in vivo* experiments with Rank-Venus mice, analyzed the data, and wrote the manuscript. MM performed *in vivo* experiments with aged mice, analyzed the data, and wrote the manuscript. GA contributed critical reagents used in this study and provided critical experimental advice. JZ-P provided critical experimental advice and edited the manuscript. All authors contributed to the article and approved the submitted version.

## Conflict of Interest

We have submitted a patent describing the method of producing and using Stemregenin-expanded proT cells. JZ-P is a co-founder of Notch Therapeutics.
