# Supplementary material for: Thymic Engraftment by in vitro-Derived Progenitor T Cells in Young and Aged Mice
Source: Front Immunol. 2020 Aug 18;11:1850. doi: 10.3389/fimmu.2020.01850 (PMC7462002; doi:10.3389/fimmu.2020.01850)
Supplement: Supplementary file 1 [file Data_Sheet_1.docx]

# Supplemental Methods

**Umbilical Cord Blood Samples**

Human UCB samples were obtained and HSPC containing fractions were purified using CD34 progenitor cell isolation kits (Miltenyi Biotec) following manufacturers protocols as previously described (1) in accordance with approved guidelines established by the Research Ethics Board of Sunnybrook Health Sciences Centre.

**Mice**

NOD.cg-PrkdcscidIL2rgtm/Wjl/Sz (NOD-*scid* IL2Rγ^null^, NSG) mice were purchased from Jackson Laboratory (Bar Harbor, ME) and housed and bred in a pathogen-free facility. RANK-Venus hSirpαTgRag^-/-^γc^null^ mice (RV-SRG) were generated by crossing RANK Venus BAC transgenic mice expressing the fluorescent protein Venus under the control elements of the murine *Tnfrsf11a* gene (2) with immunodeficient hSirpαTgRag^-/-^γc^null^ mice (The Jackson Laboratory). C57BL/6 (B6 CD45.2) and congenic B6.SJL-Ptprca Pepcb/BoyJ (B6 CD45.1) mice were purchased from The Jackson Laboratory (stock numbers 000664 and 002014, respectively). Young (8 to 12 weeks), and in-house aged (18-20 months) cohorts of mice were used. To generate Green fluorescent protein (GFP^+^) hematopoietic cells, ROSA26-rtTA transgenic mice (3) were bred to Vav-iCre transgenic mice (4) to establish VaviCre-ROSA26rtTA mice on the B6 CD45.2 background. GFP was expressed in hematopoietic cells of these mice upon Cre-dependent removal of a loxP-stop-loxP cassette within the ROSA26 locus. All mice were bred and maintained at Sunnybrook Health Sciences Centre, and all animal procedures were approved by the Sunnybrook Health Sciences Centre Animal Care Committee.

**Progenitor T cell co-cultures with OP9-DL4 cells**

Human HSC/OP9-DL4 cell co-cultures were maintained in α-MEM medium supplemented with 20% fetal bovine serum (Gibco), plus 100U/mL penicillin/streptomycin (OP9-media) as previously described (1). Every 5 days, co-cultures were transferred onto a fresh confluent monolayer of OP9-DL4 cells in OP9-media supplemented with recombinant human cytokines Flt-3L (5ng/mL), IL-7 (5ng/mL) and SCF (50ng/mL) (Miltenyi). Co-cultures were carried out for 10-12 days and human proT cells (CD34^+^CD7^+^) were flow cytometrically sorted if applicable.

For aged mouse experiments, LSK/OP9-DL4 cell co-cultures were maintained as previously described (5) with some modifications. Briefly, BM cells were harvested from B6 CD45.1 mice and enriched for CD117^+^ (ckit^+^) cells using anti-CD117-MACS beads (Miltenyi) according to manufacturer’s instructions. The CD117-enriched cells were subsequently labelled with FITC-conjugated antibodies against lineage markers (CD3, CD4, CD8, CD11b, CD19, NK1.1, Gr1, Ter119), CD117-APC and Sca-1-PE (all from BioLegend) and sorted for LSK cells using cell sorter FACSAria Fusion (BD Biosciences). 50,000 LSK cells were seeded per plate of OP9-DL4 cells at 80-90% confluency in 15 cm dishes and incubated α-Minimum Essential Medium Eagle (supplemented with 5% FBS and 1% Penicillin/streptomycin) (Gibco) in the presence of 5 ng/ml Flt-3L (Miltenyi Biotec) and 1 ng/ml IL-7 (Miltenyi). On days 5 and 8 after the start of co-cultures, old media was replaced with fresh media. The cultures were harvested on day 10. To enrich for CD25^+^ cells, the cells were labelled with anti-CD25-APC (Bio-Legend) followed by anti-APC-MACS beads (Miltenyi) incubation and purification according to manufacturer’s instructions.

**Hybrid Human-Mouse FTOC**

Fetal thymuses were isolated from embryos of timed pregnant NSG mice (The Jackson Laboratory) at day 15 of gestation. Human proT subsets were sorted from day 10-12 human HSC/OP9-DL4 co-cultures, and incubated with or without 5µg/mL OPG-Fc (R&D Systems) for 1 hour. Subsequently, proT-cells were placed into hanging drops with the fetal thymuses in Terasaki wells for 24-26 hours, followed by FTOC in the presence of absence of OPG-Fc for 5 days. Individual thymic lobes were homogenized in Trizol reagent and prepared for QPCR analysis.

**Quantitative Real Time Reverse Transcriptase Polymerase Chain Reaction (QPCR)**

Thymuses from FTOC cultures were homogenized in Trizol reagent and reverse-transcribed using Superscript III and Oligo(dT)_12-18_ primers (Invitrogen). Diluted cDNA samples were used as templates for the QPCR reactions. Detection of QPCR was performed using SYBR Green PCR master mix (Invitrogen) on the Applied Biosystems Sequence Detection system. Relative expression of genes was calculated by the delta cycle threshold (Δ-Ct) method with the expression of β-actin as an internal reference.

**Adoptive Transfer of Progenitor T cells**

CD34^+^CD7^+^ cells were sorted from day 10-12 human HSC/OP9-DL4 cell co-cultures, resuspended in a mixture containing rhIL-7 (0.5μg) and an IL-7 antibody M25 (2.5μg), and injected intrahepatically (30μL/mouse) into non-irradiated 2-5 day old RV-SRG neonates. Thymuses were harvested two weeks post-injection, and TECs were analyzed as previously described using flow cytometry (2, 6).

For aged mouse experiments, B6 CD45.2 hosts received 1.025 Gy total body irradiation using a Cs137 source gamma irradiator. After 4-6 hours, all mice were intravenously injected with 1×10^6^ B6 GFP^+^ BM-extracted cells from VaviCre-ROSA26rtTA mice. Some mice also received 6×10^6^ CD25-enriched proT cells derived from B6 CD45.1 mice, at 99% purity.

**Flow Cytometric Analysis**

To analyze the expression of LTβR, RANKL and CD40L, Lineage^-^CD34^+^CD38^-/lo^ human HSCs from UCB or CD34^+^CD7^+^ proT cells from a day 10-12 HSC/OP9DL4 co-culture were stained with anti-human RANKL-PE for analysis of cell surface RANKL expression. For detection of LTαβ and CD40L, LTβR-Fc and CD40-Fc fusion proteins were used, followed by a secondary anti-human IgG-PE antibody.

For aged mouse experiments, single cell suspensions were prepared for thymus by mashing followed by filtering through cell strainers in Hanks’ Balanced Salt Solution supplemented with 1% bovine serum albumin and 2 mM EDTA. a-MEM. Single-cell suspensions were labelled with fluorescently-conjugated antibodies purchased from BioLegend as follows: CD45.1-Percp/Cy5.5, CD45.2-APC/Cy7, CD4-Alexafluor 700, CD8-PE/Cy7, CD44PE, CD25-APC. Flow cytometry was performed on an LSR II (BD Biosciences), and data were analyzed with FlowJo software version 9.7.6.

**Statistical Analysis**

To determine statistical significance between non-injected and proT-injected RV-SRG mice-derived TEC populations, or BM and BM+proT adoptive transfer experiments, a two-tailed unpaired student’s t-test was performed using R software. Kruskal-Wallis one-way analysis of variance with post-hoc Dunn’s test was used to determine significant differences across multiple groups for the FTOC experiments. All data are represented as mean ± SEM, with asterisks representing statistical significance (*p < 0.05, **p < 0.01).

**References**

1. G. Awong, E. Herer, C. D. Surh, J. E. Dick, R. N. La Motte-Mohs and J. C. Zuniga-Pflucker: Characterization in vitro and engraftment potential in vivo of human progenitor T cells generated from hematopoietic stem cells. *Blood*, 114(5), 972-82 (2009) doi:10.1182/blood-2008-10-187013

2. S. Baik, E. J. Jenkinson, P. J. Lane, G. Anderson and W. E. Jenkinson: Generation of both cortical and Aire(+) medullary thymic epithelial compartments from CD205(+) progenitors. *Eur J Immunol*, 43(3), 589-94 (2013) doi:10.1002/eji.201243209

3. G. Belteki, J. Haigh, N. Kabacs, K. Haigh, K. Sison, F. Costantini, J. Whitsett, S. E. Quaggin and A. Nagy: Conditional and inducible transgene expression in mice through the combinatorial use of Cre-mediated recombination and tetracycline induction. *Nucleic Acids Res*, 33(5), e51 (2005) doi:10.1093/nar/gni051

4. M. Stadtfeld and T. Graf: Assessing the role of hematopoietic plasticity for endothelial and hepatocyte development by non-invasive lineage tracing. *Development*, 132(1), 203-13 (2005) doi:10.1242/dev.01558

5. M. Mohtashami, D. K. Shah, H. Nakase, K. Kianizad, H. T. Petrie and J. C. Zuniga-Pflucker: Direct comparison of Dll1- and Dll4-mediated Notch activation levels shows differential lymphomyeloid lineage commitment outcomes. *J Immunol*, 185(2), 867-76 (2010) doi:10.4049/jimmunol.1000782

6. S. Baik, M. Sekai, Y. Hamazaki, W. E. Jenkinson and G. Anderson: Relb acts downstream of medullary thymic epithelial stem cells and is essential for the emergence of RANK(+) medullary epithelial progenitors. *Eur J Immunol*, 46(4), 857-62 (2016) doi:10.1002/eji.201546253
